# Amantadine-Induced Patulous Eustachian Tubes in Parkinson's Disease

**DOI:** 10.1155/2013/426413

**Published:** 2013-11-20

**Authors:** J. T. Boyd, D. A. Silverman

**Affiliations:** ^1^Department of Neurological Sciences, College of Medicine, University of Vermont, UHC-Arnold 2, 1 South Prospect Street, Burlington, VT 05401, USA; ^2^Department of Surgery (Otolaryngology), College of Medicine, University of Vermont, Burlington, VT 05401, USA

## Abstract

Patulous Eustachian tube (PET) is a common condition that produces symptoms of aural fullness and autophony. We describe a Parkinson's disease (PD) patient that experienced a reversible bilateral patulous (hyperpatent) Eustachian tube syndrome induced by treatment with amantadine hydrochloride. The clinical features, relevant anatomy and physiology, and associated risk factors for PET are reviewed.

## 1. Introduction

Patulous Eustachian tube (PET) is a common condition that presents with the primary complaint of distorted or echoed autophony. Autophony is the amplified auditory perception of self-generated sounds such as respiration, vocalization, and swallowing [1–3]. Additional presenting features can include a feeling of aural fullness, mild vertigo or imbalance, recurrent nasal sniffing to relieve autophony, and positional symptom improvement when supine. Persistent communication of the pharynx and middle ear occurs in the setting of impaired closure of the Eustachian tube at the nasopharyngeal orifice [4, 5]. Previously reported contributing factors include weight loss, caffeine use, pregnancy, and medications including diuretics and nasal decongestants [3–7]. Neurological disorders that have been associated with PET include motor neuron disease, multiple sclerosis, and stroke [3–8]. 

In healthy cases, the ET closes passively via recoil of the elastin hinge and force generated by the deformation of Ostmann's fat pad. Opening occurs during yawning, sneezing, and deglutition actively by contraction of the tensor veli palatini and passively by the levator veli palatini. Mucus producing goblet cells line the medial two-thirds of the ET near the pharyngeal orifice [3, 7].

## 2. Case Presentation

We report the case of a patient who developed PET induced by amantadine hydrochloride which resolved with discontinuation. The patient is a 50-year-old man with a one-year history of Parkinson's disease (Hoehn and Yahr stage 1). 

Amantadine hydrochloride was initiated and escalated to 100 mg twice daily with improvement of parkinsonism and initial absence of adverse effects. Approximately four weeks after initiation of amantadine, the patient presented with complaints of oropharyngeal dryness with constant left and intermittent right autophony. Visualization of the tympanic membrane revealed left-sided mobility in association with deep respiration, supporting a diagnosis of Eustachian tube hyperpatency. No additional pathology was identified on flexible laryngoscopic examination. 

Discontinuation of amantadine resulted in a resolution of symptoms over seven days. Amantadine rechallenge resulted in a rapid reemergence of the aforementioned symptoms. The patient elected to undergo unilateral left tube myringotomy with limited symptom relief. 

## 3. Discussion

Reversible patulous Eustachian tubes were induced by amantadine in our patient. PET is described in the literature as common and often induced by medications, though peer-reviewed reports of particular medication-induced PET are lacking. PET has not previously been reported in association with Parkinson's disease or its treatment. Amantadine is a commonly used symptomatic treatment in both early and advanced PD. Multiple mechanisms of action are proposed including NMDA glutamate antagonism and enhanced release/reduced reuptake of dopamine [9]. Dry mouth, constipation, and other peripheral anticholinergic side effects are commonly encountered with amantadine. Previous investigations suggest that these may be related to a reduced cholinergic release rather than receptor antagonism [9]. Other medications previously associated with PET include diuretics and decongestants [4, 5]. Given the simultaneous complaints of oral dryness and known anticholinergic side effects, amantadine may have reduced mucus production/increased viscosity to promote PET. Weight loss, altered voluntary and involuntary muscle activation, and mucosal dryness are common to both PET and PD. Thus, the recognition of a potential association between PET and amantadine represents an important clinical observation.
